# A98 PATIENT NAVIGATOR PROGRAMS FOR CHRONIC GASTROINTESTINAL DISORDERS: A SCOPING REVIEW

**DOI:** 10.1093/jcag/gwae059.098

**Published:** 2025-02-10

**Authors:** A P Souza Lira, J Osei, R Sanderson, C D McIlduff, S Fowler, J Peña-Sánchez

**Affiliations:** Department of Community Health and Epidemiology, University of Saskatchewan, Saskatoon, SK, Canada; Department of Community Health and Epidemiology, University of Saskatchewan, Saskatoon, SK, Canada; James Smith Cree Nation, James Smith Cree Nation, SK, Canada; Department of Community Health and Epidemiology, University of Saskatchewan, Saskatoon, SK, Canada; Department of Community Health and Epidemiology, University of Saskatchewan, Saskatoon, SK, Canada; Department of Community Health and Epidemiology, University of Saskatchewan, Saskatoon, SK, Canada

## Abstract

**Background:**

Individuals living with chronic gastrointestinal disorders (CGIDs), including inflammatory bowel disease (IBD), diverticulitis (DI), gastroesophageal reflux disease (GERD), and undiagnosed gastrointestinal complaints (UGCs), could encounter healthcare barriers, such as delays in diagnosis, frequent hospital visits, complex treatment plans, financial concerns, transportation difficulties, among others. Patient navigator programs (PNPs) have emerged as a potential solution to enhance healthcare access and improve care for chronic diseases, including CGIDs.

**Aims:**

To synthesize available literature on PNPs for CGIDs, describing their focus and impacts.

**Methods:**

A scoping review was completed across seven databases: MEDLINE, EMBASE, CINAHL, SCOPUS, Web of Science, Global Health, and Public Health. Qualitative, quantitative, mixed-method studies and abstracts published between 2012 and 2022 were included in this review. Eligibility criteria included publications in English focused on PNPs for CGIDs. Two independent reviewers screened titles, abstracts, and available full texts. The included publications were classified and summarized.

**Results:**

11 publications were included in the review; the majority were from the United States (n=5). There were two publications from Australia, two from Canada, one from Belgium, and one from Chile. There were nine full papers and two conference abstracts. By study design, there were four retrospective cohorts, three cross-sectionals, two case-control, one prospective cohort, and one study protocol for a randomized controlled trial. The 11 publications described 10 PNPs for CGIDs, which were implemented between 2008 and 2022 in urban areas, and all were linked to hospital clinics or tertiary referral centres. Nurses led ten PNPs, and eight programs were for IBD care. The other three programs were for persons with GERD, DI, or UGCs. These publications reported the positive impact of PNPs on multiple healthcare utilization and patient outcomes, including more timely access to appointments and diagnostic tests, decreased hospital admission rates, fewer emergency department visits, high user satisfaction levels, reductions in no-show rates for gastroenterology appointments and direct healthcare costs, and a reduced need for pain medications post-hospital discharge.

**Conclusions:**

This scoping review identified 10 PNPs for CGIDs around the world; the majority of these PNPs were led by nurses and designed for IBD care. PNPs for CGIDs have evidence of multiple benefits for individuals and the healthcare system. Further research could explore the impact on patient outcomes of PNPs for CGIDs living in rural and remote locations, as well as their impact on individuals with CGIDs belonging to equity-deserving groups.

**Funding Agencies:**

None

